# TNF, but not hyperinsulinemia or hyperglycemia, is a key driver of obesity‐induced monocytosis revealing that inflammatory monocytes correlate with insulin in obese male mice

**DOI:** 10.14814/phy2.13937

**Published:** 2018-12-09

**Authors:** Jessica A. Breznik, Avee Naidoo, Kevin P. Foley, Christian Schulz, Trevor C. Lau, Dessi Loukov, Deborah M. Sloboda, Dawn M. E. Bowdish, Jonathan D. Schertzer

**Affiliations:** ^1^ Department of Pathology and Molecular Medicine McMaster University Hamilton Canada; ^2^ McMaster Immunology Research Centre McMaster University Hamilton Canada; ^3^ Michael G. DeGroote Institute for Infectious Disease Research McMaster University Hamilton Canada; ^4^ Department of Biochemistry and Biomedical Sciences McMaster University Hamilton Canada; ^5^ Farncombe Family Digestive Health Research Institute McMaster University Hamilton Canada; ^6^ Department of Obstetrics and Gynecology and Pediatrics McMaster University Hamilton Canada

**Keywords:** inflammation, insulin, monocyte, obesity, TNF

## Abstract

Inflammation contributes to obesity‐related hyperinsulinemia and insulin resistance, which often precede type 2 diabetes. Inflammation is one way that obesity can promote insulin resistance. It is not clear if the extent of obesity, hyperinsulinemia, or hyperglycemia, underpins changes in cellular immunity during diet‐induced obesity. In particular, the requirement for obesity or directionality in the relationship between insulin resistance and monocyte characteristics is poorly defined. Inflammatory cytokines such as tumor necrosis factor (TNF) can contribute to insulin resistance. It is unclear if TNF alters monocytosis or specific markers of cellular immunity in the context of obesity. We measured bone marrow and blood monocyte characteristics in WT and TNF ^−/−^ mice that were fed obesogenic, high fat (HF) diets. We also used hyperglycemic Akita mice and mice implanted with insulin pellets in order to determine if glucose or insulin were sufficient to alter monocyte characteristics. We found that diet‐induced obesity in male mice increased the total number of monocytes in blood, but not in bone marrow. Immature, inflammatory (Ly6C^high^) monocytes decreased within the bone marrow and increased within peripheral blood of HF‐fed mice. We found that neither hyperinsulinemia nor hyperglycemia was sufficient to induce the observed changes in circulating monocytes in the absence of diet‐induced obesity. In obese HF‐fed mice, antibiotic treatment lowered insulin and insulin resistance, but did not alter circulating monocyte characteristics. Fewer Ly6C^high^ monocytes were present within the blood of HF‐fed TNF ^−/−^ mice in comparison to HF‐fed wild‐type (WT) mice. The prevalence of immature Ly6C^high^ monocytes in the blood correlated with serum insulin and insulin resistance irrespective of the magnitude of adipocyte or adipose tissue hypertrophy in obese mice. These data suggest that diet‐induced obesity instigates a TNF‐dependent increase in circulating inflammatory monocytes, which predicts increased blood insulin and insulin resistance independently from markers of adiposity or adipose tissue expansion.

## Introduction

Obesity is associated with chronic, low‐grade systemic inflammation that also impacts endocrine control of metabolism (McPhee and Schertzer 2015). This metabolic inflammation (or metaflammation) can impair insulin action in tissues that participate in blood glucose control. The consequent insulin resistance is often matched by hyperinsulinemia, which independently promotes obesity and insulin resistance that generally precedes hyperglycemia and type 2 diabetes (T2D) (Mehran et al. 2012). In combination with other environmental and genetic factors, prolonged hyperinsulinemia, insulin resistance and metaflammation during obesity, coupled with insufficient insulin secretion, can lead to elevated blood glucose and the development of T2D (Ashcroft and Rorsman 2012).

Obesity alters endocrine and immunological communication between cells and tissues. For example, increased inflammatory mediators in adipose tissue contribute to reduced insulin sensitivity within adipocytes and other tissues that help control blood glucose. Endocrine and paracrine actions of cytokines, chemokines and adipokines produced by expanding adipose tissue and tissue‐localized immune cells during obesity contribute to local and systemic inflammation and the development of insulin resistance (Weisberg et al. 2006; Lumeng et al. 2007a,b). The molecular mechanisms that connect insulin resistance and inflammatory cytokines/chemokines are widely studied. Comparatively little is known about how cellular immunity relates to compartmentalized, tissue‐specific inflammatory cues and insulin resistance during obesity.

Accumulation of pro‐inflammatory polarized macrophages within metabolic tissues is a key component of metabolic inflammation, as ablation or inhibition of macrophage inflammatory signaling can attenuate obesity‐induced insulin resistance in adipose and liver tissue (Arkan et al. 2005; Cai et al. 2005; Han et al. 2013; Revelo et al. 2016; Desai et al. 2017). Adipose tissue macrophages in particular are derived from local proliferation, resident hematopoietic stem cells, and infiltration of peripheral blood monocytes (macrophage precursors) (Weisberg et al. 2003; Xu et al. 2003; Oh et al. 2012; Amano et al. 2014; Luche et al. 2017). Murine monocytes can be divided into subsets by their surface expression of the glycoprotein Ly6C: Ly6C^−^ (also referred to as Ly6C^low^) and Ly6C^+^ (Ly6C^int^ and Ly6C^high^) (Geissmann et al. 2003; Gordon and Taylor 2005). Ly6C^high^ monocytes mature into Ly6C^−^ patrolling monocytes in the steady state but migrate to sites of acute inflammation during infection via CCL2/CCR2‐dependent chemotaxis, extravasate into tissues, and differentiate into macrophages (Geissmann et al. 2003; Gordon and Taylor 2005; Serbina and Pamer 2006; Tsou et al. 2007). The pro‐inflammatory cytokine TNF contributes to monocyte migration during infection by promoting CCR2 expression (Boekhoudt et al. 2003). CCR2^−/−^ mice are protected against obesity‐induced macrophage accumulation, inflammation, hyperinsulinemia, and insulin resistance (Chen et al. 2005; Weisberg et al. 2006; Ito et al. 2008). TNF is produced by tissue‐localized macrophages in obese mice, and its neutralization improves insulin sensitivity (Hotamisligil et al. 1995; Uysal et al. 1997). Thus, we hypothesized that during diet‐induced obesity there is a TNF‐dependent egress of Ly6C^high^ monocytes from bone marrow into circulation, these monocytes contribute to tissue‐associated macrophage accumulation, and they are associated with metainflammation and insulin resistance.

We initially assessed the effects of diet‐induced obesity on monocyte prevalence in bone marrow and blood and their expression of inflammatory and maturity markers (Ly6C, F4/80) in wild‐type (WT) male mice. We found that monocyte reprogramming during obesity begins in the bone marrow and is marked by increased circulating Ly6C^−^, Ly6C^int^, and Ly6C^high^ monocytes in HF‐fed mice. Using TNF^−/−^ male mice as a model of reduced systemic inflammation, we demonstrated that despite diet‐induced obesity in TNF^−/−^ mice there was a reduction in circulating Ly6C^high^ inflammatory monocytes and macrophage accumulation in adipose tissue in comparison to WT mice. More importantly, this discovery allowed for an assessment of insulin resistance characteristics in obese mice that had different levels of circulating Ly6C^high^ monocytes. We determined that during diet‐induced obesity the TNF‐dependent prevalence of blood monocytes, and inflammatory Ly6C^high^ monocytes in particular, were better predictors of indices of insulin resistance than body weight or parameters of adiposity, such as adipocyte size. We found no evidence that this relationship is bidirectional since manipulation of insulin levels, insulin resistance, or blood glucose did not alter monocyte characteristics.

Importantly, obesity‐induced increases in circulating Ly6C^high^ monocytes directly correlated with blood insulin levels irrespective of adipose tissue/cell expansion in obese mice.

## Materials and Methods

### Animals

WT C57BL/6J male mice and TNF^−/−^ male mice on a C57BL/6J background were originally purchased from The Jackson Laboratory (WT no. 00064; TNF^−/−^ no. 003008; Bar Harbor, ME) and bred at the McMaster Central Animal Facility (Hamilton, ON, Canada), as described (Zganiacz et al. 2004; Puchta et al. 2016). Heterozygous C57BL/6‐Ins2^Akita^/J (Akita^+/−^) male mice were purchased from The Jackson Laboratory (no. 003548). All animals were housed under specific pathogen‐free conditions with a 12‐hour light/dark cycle. Diets were manipulated at 8 weeks of age. WT and TNF^−/−^ male mice (not littermates) were allocated to either *ad libitum* standard chow diet (18% kcal from fat; Envigo Teklad Diets 7913, Madison, WI) or an obesogenic, low fiber, high fat (HF) diet (60% kcal from fat; Research Diets Inc. D12492, New Brunswick, NJ) for 18–24 weeks. Akita^+/−^ male mice and WT male mice were used for hyperglycemia experiments at 8 weeks of age. WT male mice used for hyperinsulinemia experiments were maintained on an *ad libitum* standard chow diet. To induce hyperinsulinemia, subcutaneous implantation of insulin pellets, or sham surgery was performed on WT male mice at 10–12 weeks of age as recommended by the manufacturer (~0.1U/day release; LinBit for Mice Pr‐1‐B, LinShin Canada Inc., Toronto, ON, Canada). WT male mice used for antibiotic microbiota depletion experiments were maintained on HF diet for 20 weeks prior to antibiotic treatment, which consisted of 1 g/L ampicillin (Sigma A6140) and 0.5 g/L neomycin (Sigma N1878) in drinking water. Antibiotics were changed every 2 days over 4 weeks. All experiments were performed in accordance with Institutional Animal Utilization Protocols approved by McMaster University's Animal Research Ethics Board following the recommendations of the Canadian Council for Animal Care. Data from each individual mouse are indicated by a single symbol in all figures.

### Metabolic assessments

Mice were fasted for 6 h with *ad libitum* water. Blood glucose and insulin were measured to calculate HOMA‐IR as we have done previously (Cavallari et al. 2017). Fasting blood glucose was measured via tail vein using the Accu‐Chek Inform II system glucometer and test strips (Roche Diagnostics, Mississauga, ON, Canada). Blood (50 *μ*L) was collected via tail vein, incubated at room temperature for 20 min, and spun at 7500*g* for 5 min at 4°C. Insulin was quantified in serum by ELISA according to the manufacturer's instructions (Fig. 3 ‐ AIS Toronto Biosciences 32270, Toronto, ON, Canada; Figs. 4 and 6 ‐ EMD Millipore EZRMI‐13K, Billerica, MA).

### Flow cytometry

Bone marrow flushed from a femur and disrupted with an 18‐gauge needle, or peripheral blood collected retro‐orbitally (100 *μ*L), was analyzed by flow cytometry to identify monocyte populations, as previously published (Puchta et al. 2016). Monoclonal antibodies with the following conjugated fluorophores were used in this study with isotype controls: CD3 (Alexa Fluor 700), CD11b (PE‐Cy7), CD19 (Alexa Fluor 700), CD45 (eFluor 450), F4/80 (APC), IL‐6 (PerCP‐eFluor 710), Ly6G (Alexa Fluor 700), and NK1.1 (Alexa Fluor 700), all from eBioscience (San Diego, CA), as well as CCR2 (PE; R&D Systems, Minneapolis, MA) and Ly6C (FITC; BioLegend, San Diego, CA). Intracellular staining was performed on blood in unstimulated conditions and following stimulation with lipopolysaccharide (200 ng/mL) in complete RPMI supplemented with 10% fetal calf serum and 2x Protein Transport Inhibitor (eBioscience 00‐4980‐03). Samples were initially surface stained with antibodies and then intracellular staining was performed after 30 min permeabilization at room temperature with 1x Intracellular Staining Permeabilization Wash Buffer (BioLegend 421002) as previously described (Puchta et al. 2016). Flow cytometry was performed on a BD Biosciences LSRFortessa and analyzed using the FlowJo v9 software (Tree Star). Total cell counts were determined with CountBright Absolute Counting Beads (Life Technologies C36950).

### Immunohistochemistry

Adipose tissue macrophages were analyzed as published (Denou et al. 2015). Epididymal fat pads were fixed in 10% formalin at room temperature and embedded in paraffin. 5‐*μ*m sections cut at 50‐*μ*m intervals were mounted on positively charged glass slides, deparaffinized in xylene, treated with proteinase K, and stained with an anti‐F4/80 (1:500) monoclonal antibody (Bio‐Rad Antibodies MCA‐497) on the Leica Bond RX automated staining system. Adipocyte size and total number of F4/80‐expressing cells (macrophages) were assessed using ImageJ (Schneider et al. 2012).

### Statistical analyses

Monocyte population prevalence and phenotype differences (for individual monocyte populations) were evaluated with the Mann‐Whitney U test or Student's *t* test according to normality by the D'Agostino and Pearson omnibus test. Differences in HF‐fed TNF^−/−^ and WT adipose tissue macrophages, adipocyte cross‐sectional area, and fasting blood glucose were evaluated with the Mann‐Whitney U test or Student's *t* test according to normality. Body mass and metabolic parameter comparisons by genotype and diet were performed by two‐way ANOVA with Tukey's post hoc test. Correlation analyses between adipose tissue characteristics, monocytes, and metabolic parameters, were performed using Spearman's rank correlation rho or Pearson correlation according to normality. Data were analyzed with GraphPad Prism version 6 (GraphPad Software, La Jolla, CA). A *P* < 0.05 was considered statistically significant.

## Results

### Diet‐induced obesity alters monocyte populations in bone marrow and blood

**Figure 1:**
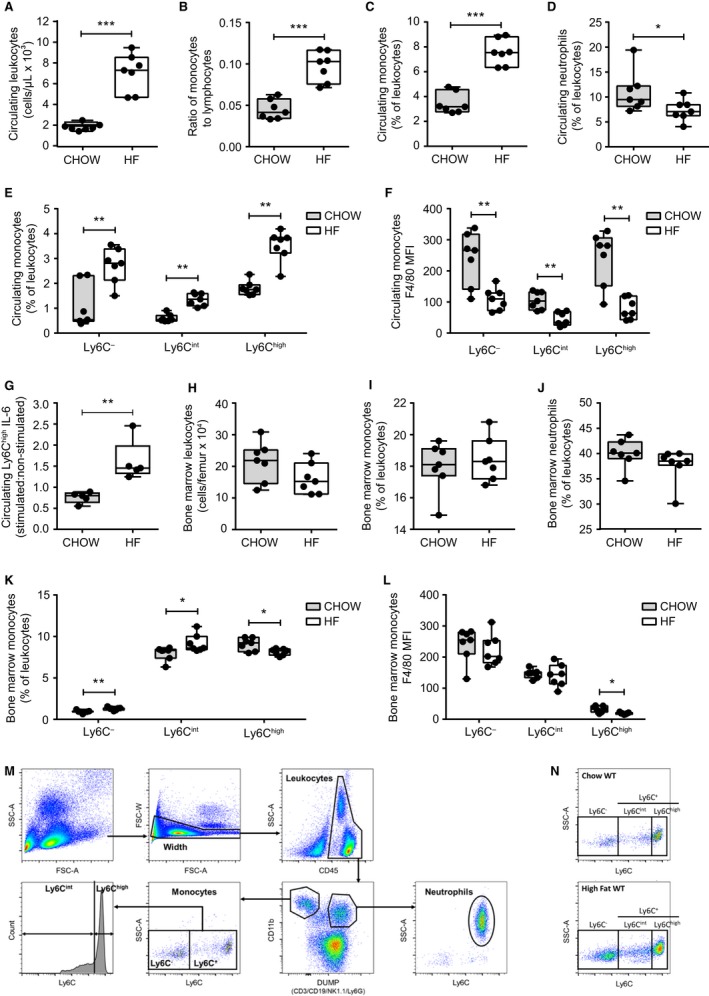
Diet‐induced obesity increases circulating monocyte populations. Peripheral whole blood was analyzed in chow‐fed (*n* = 7) and HF‐fed (*n* = 7) WT male mice after 18 weeks diet allocation. (A) absolute count of circulating leukocytes (CD45^+^ cells). (B) proportion of monocytes to lymphocytes. (C) circulating monocytes. (D) circulating neutrophils. (E) proportions of Ly6C^−^, Ly6C^int^, and Ly6C^high^ subsets of monocytes in chow‐fed and HF‐fed mice. (*F*) expression of maturity marker F4/80 in circulating monocyte subsets from chow‐fed and HF‐fed mice. (G) IL‐6 levels within Ly6C^high^ monocytes in response to LPS stimulation in chow‐fed (*n* = 5) and HF‐fed (*n* = 5) mice. (H) absolute count of bone marrow leukocytes. (I) bone marrow monocytes. (J) bone marrow neutrophils. (K) proportions of Ly6C^−^, Ly6C^int^, and Ly6C^high^ subsets of monocytes. (L) expression of maturity marker F4/80 in bone marrow monocyte subsets. Each data point indicates a single mouse. Statistical significance was determined by Mann‐Whitney tests. Data are presented as box and whiskers plots, minimum to maximum, where the center line represents the median. *P ≤* *0.05, **P ≤* *0.01, ***P ≤* *0.001. MFI – Geometric Mean Fluorescence Intensity. (M) flow cytometry gating strategy for the identification of leukocytes, neutrophils, and monocytes (bone marrow‐resident and circulating). Representative images from a blood sample are shown. A width gate was created to exclude cell aggregates. CD45^+^ cells (leukocytes) were first gated. Subsequently, CD11b^+/−^ and AF700^+/−^ population gating allowed separation of CD11b^+^Ly6G^−^ (monocytes), CD11b^+^Ly6G^+^ (neutrophils), and CD11b^mid/−^ CD3^+^ CD19^+^ NK1.1^+^ (lymphocytes: T cells, B cells, NK cells) cell populations. The CD45^+^ CD11b^+^Ly6G^+^Ly6C^+^ SSC ^high^ cells were identified as neutrophils and the CD45^+^ CD11b^+^Ly6G^−^Ly6C^+^ SSC ^low^ monocyte cells were divided into subsets by their expression of Ly6C: Ly6C^−^, Ly6C^int^, and Ly6C^high^. (N) Representative flow plots of monocyte populations in chow‐fed and HF‐fed wild‐type male mice.

Leukocytosis, an increase in white blood cells, occurs in obese and/or diabetic humans (Kullo et al. 2002; Ohshita et al. 2004; Tong et al. 2004). More recently, an elevation in circulating monocytes (monocytosis) has been associated with poorly controlled T2D, cardiovascular disease, and increased adiposity (Poitou et al. 2011; Barrett et al. 2017). Similar to Nagareddy and colleagues (Nagareddy et al. 2014), we found that WT male mice fed a HF diet for 18 weeks had a significant increase in circulating leukocytes in peripheral whole blood (Fig. 1A). HF‐fed mice had an elevated ratio of circulating monocytes to lymphocytes in comparison to chow‐fed control mice (Fig. 1B), due to a disproportionate increase in the prevalence of circulating monocytes (Fig. 1C) and a small decrease in the prevalence of circulating neutrophils (Fig. 1D). To further investigate the effect of diet‐induced obesity on monocyte characteristics, we assessed their prevalence and expression of surface markers of inflammation (Ly6C) and maturity (F4/80). When we assessed the circulating monocyte subsets according to their expression of Ly6C it was apparent that male mice on a HF diet had elevated circulating populations of Ly6C^−^, Ly6C^int^, and Ly6C^high^ monocytes (Fig. 1E). All monocyte subsets were increased more than twofold (as a proportion of total leukocytes) in the circulation of HF‐fed mice. Absolute cell counts were also elevated in HF‐fed mice (data not shown). Ly6C^high^ monocytes were significantly less mature (decreased F4/80 expression) in HF‐fed mice (Fig. 1F) and Ly6C^high^ had higher surface expression of CCR2 in comparison to the other monocyte subsets (data not shown) (Chen et al. 2005; Tsou et al. 2007). Intracellular staining also indicated that circulating Ly6C^high^ monocytes from HF‐fed mice produced higher levels of the pro‐inflammatory cytokine IL‐6 in response to LPS stimulation (Fig. 1G). Thus, diet‐induced obesity in male mice is accompanied by an increase in circulating, immature inflammatory Ly6C^high^ monocytes. It was previously demonstrated that HF‐fed mice have increased proliferation of the hematopoietic common myeloid progenitors within bone marrow (Nagareddy et al. 2014). We determined that leukocyte numbers and monocyte prevalence, as well as the prevalence of neutrophils, were unchanged within bone marrow of HF‐fed mice (Fig. 1H–J). However, Ly6C^−^ and Ly6C^int^ monocytes were elevated whereas Ly6C^high^ monocytes were decreased in the bone marrow of HF‐fed mice (Fig. 1K). In addition, bone marrow‐resident Ly6C^high^ monocytes in HF‐fed mice had lower expression of the F4/80 maturity marker (Fig. 1L). These data are consistent with the concept that diet‐induced obesity promotes egress of immature, inflammatory Ly6C^high^ monocytes from the bone marrow into circulation. The flow cytometry gating strategy is illustrated in Figure 1M, and representative plots of monocyte populations in chow‐fed and HF‐fed mice are shown in Figure 1N. We subsequently assessed whether specific metabolic factors (such as glucose, insulin, insulin resistance or extent of obesity/adiposity) were associated with elevated circulating Ly6C^high^ monocytes.

### Elevated blood glucose or insulin alone is insufficient to alter Ly6C^high^ monocytes

**Figure 2:**
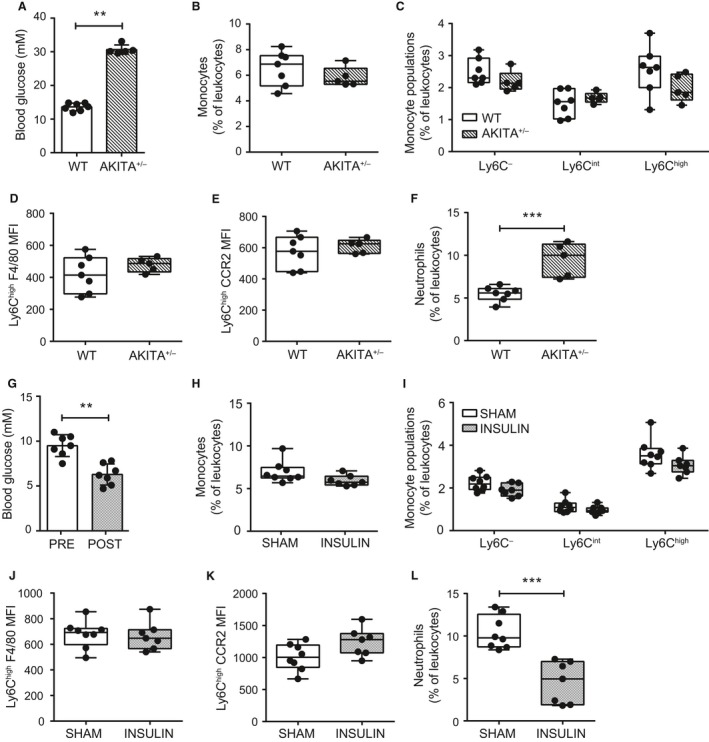
Ly6C^high^ prevalence and phenotype are independent of glucose and insulin in absence of obesity. Peripheral blood monocytes were assessed in chow fed, male WT (*n* = 7) and Akita^+/−^ (*n* = 5) mice. (A) random fed blood glucose in WT and Akita^+/−^ mice. (B and C) total monocytes and Ly6C^−^, Ly6C^int^, and Ly6C^high^ monocyte subsets as a percentage of total leukocytes (CD45^+^ cells) in WT and Akita^+/−^ mice. (D and E) Ly6C^high^ monocyte F4/80 and CCR2 surface expression in WT and Akita^+/−^ mice. (F) total neutrophils as a percentage of total leukocytes in WT and Akita^+/−^ mice. Effects of hyperinsulinemia on circulating monocytes were assessed in peripheral blood of sham (*n* = 8) and insulin pellet implanted (*n* = 7) chow‐fed, WT male mice. (G) random fed blood glucose preimplantation and 2‐weeks post‐insulin pellet implantation. (H and I) total monocytes and Ly6C‐expressing monocyte subsets as a percentage of total leukocytes (CD45^+^ cells) in mice 2 weeks after sham and post‐insulin pellet implantation. (J and K) Ly6C^high^ monocyte F4/80 and CCR2 surface expression. (L) total neutrophils as a proportion of total leukocytes in mice 2 weeks after sham and post‐insulin pellet implantation. Each data point indicates a single mouse. Two‐tailed Mann‐Whitney U tests were used to assess statistical significance between diet groups. Data are presented as box and whiskers plots, minimum to maximum, where the center line represents the median. **P ≤* *0.01, ***P ≤* *0.001. MFI: Geometric Mean Fluorescence Intensity.

The HF diet model used leads to increased adiposity, blood glucose, insulin, and insulin resistance (Cavallari et al. 2017). We next tested if elevation of blood glucose or insulin alone could increase circulating inflammatory monocytes. Chow‐fed Akita^+/−^ mice (C57BL/6‐Ins2^Akita^/J) were used to model diabetes to test the effect of hyperglycemia on monocyte characteristics, in the absence of obesity (Yoshioka et al. 1997; Gurley et al. 2006). Random fed blood glucose was significantly elevated in chow‐fed Akita^+/−^ mice in comparison to chow‐fed WT mice (Fig. 2A). No differences were identified in the percentage of circulating total monocytes or monocyte subsets (Fig. 2B and C). Ly6C^high^ monocyte surface expression of F4/80 was unchanged in hyperglycemic Akita^+/−^ mice (Fig. 2D). CCR2 expression was also unchanged in hyperglycemic Akita^+/−^ mice (Fig. 2E). However, hyperglycemic Akita^+/−^ mice had significantly elevated neutrophils in circulation (Fig. 2F). We next tested the effects of short‐term hyperinsulinemia that was sustained for 2 weeks after implanting slow release insulin pellets in WT chow‐fed mice. The increased insulin load reduced random fed blood glucose (Fig. 2G), but monocyte percentages were similar between sham and insulin pellet implanted mice (Fig. 2H and I). Expression of F4/80 and CCR2 on Ly6C^high^ monocytes was also unchanged in mice with insulin pellets (Fig. 2J and K). To contrast, we observed that mice implanted with insulin pellets had decreased circulating neutrophils (Fig. 2L). These results suggest that overt hyperglycemia, and (short‐term) chronic hyperinsulinemia are not sufficient to account for the increase in circulating immature, inflammatory Ly6C^high^ monocytes that we observed in HF diet‐fed mice.

### Antibiotic‐mediated lowering of glucose and insulin during obesity are insufficient to alter circulating Ly6C^high^ monocytes

**Figure 3:**
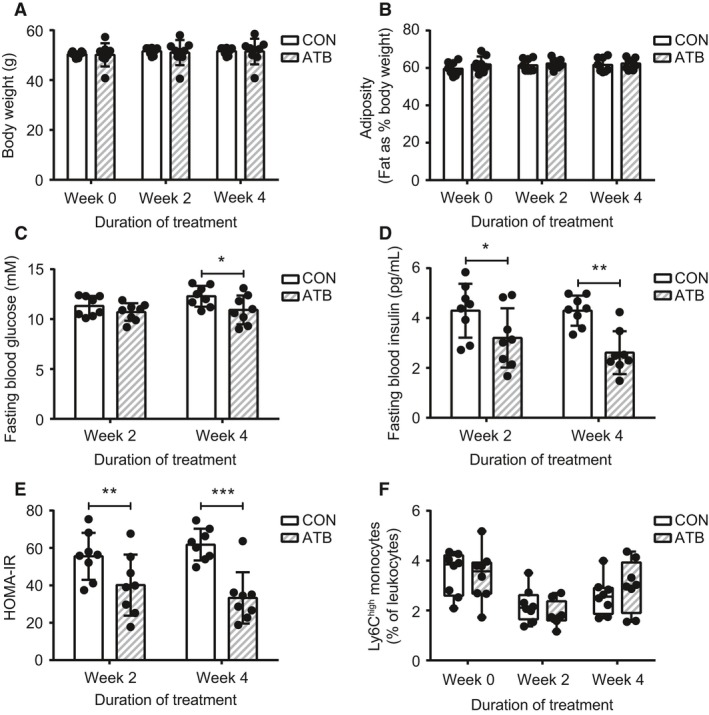
Ly6C^high^ prevalence in obesity is not altered by antibiotic‐mediated lowering of glucose and insulin. WT male mice were allocated to HF diet for 20 weeks and maintained on HF diet following allocation to usual drinking water (CON,* n* = 8) or antibiotics in drinking water (ATB,* n* = 8) for 4 weeks. Assessments occurred before allocation to antibiotic treatment (week 0) and after 2 and 4 weeks of treatment. (A) body weight. (B) adiposity. (C) fasting blood glucose. (D) fasting blood insulin. (E) HOMA‐IR. (F) circulating Ly6C^high^ monocytes as a percentage of total leukocytes (CD45^+^ cells). Each data point indicates a single mouse. Two‐tailed Student's *t* tests or Mann‐Whitney U tests were used to assess statistical significance between diet groups at each timepoint according to normality. Data are presented as bar graphs with mean ± standard deviation (*A* to *E*) or box and whiskers plots, minimum to maximum, where the center line represents the median (*F*). *P ≤* *0.05, **P ≤ 0.01, ***P ≤* *0.001.

We also considered whether circulating Ly6C^high^ monocytes can be modulated by glucose and insulin in the context of diet‐induced obesity. We used our previously established antibiotic treatment protocol in mice that modestly reduces peripheral glucose and insulin within 4 weeks (Denou et al. 2015). Mice with established diet‐induced obesity after 20 weeks of HF diet feeding were allocated to receive water as usual or drinking water supplemented with antibiotics for 4 weeks. Antibiotic treatment did not alter body weight (Fig. 3A) or adiposity (Fig. 3B). Fasting blood glucose was lower after 4 weeks of antibiotic treatment (Fig. 3C). Further, fasting blood insulin decreased at 2 weeks and 4 weeks of antibiotic treatment (Fig. 3D). Insulin resistance, measured by the homeostatic model assessment of insulin resistance (HOMA‐IR), was lower at 2 weeks and 4 weeks of antibiotic treatment (Fig. 3E). Total circulating leukocyte numbers were unaffected by antibiotic treatment (data not shown). The prevalence of circulating Ly6C^high^ monocytes was not altered by antibiotics at 2 and 4 weeks (Fig. 3F). These results were consistent with our previous data showing that manipulation of glucose and insulin is not sufficient to alter the prevalence of circulating Ly6C^high^ monocytes.

### TNF contributes to inflammatory Ly6C^high^ monocyte prevalence during diet‐induced obesity

**Figure 4:**
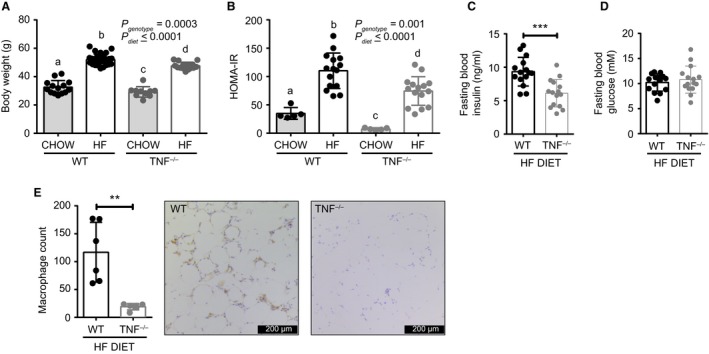
TNF contributes to insulin resistance and adipose inflammation during obesity. WT and TNF ^−/−^ male mice were allocated to a chow or HF diet for 24 weeks. Body weight (A) and HOMA‐IR (B) determined at endpoint for chow‐fed and HF‐fed WT and TNF ^−/−^ mice. Fasting blood insulin (C) and glucose (D) in HF‐fed mice WT and TNF ^−/−^ mice. (E) quantification of adipose tissue‐resident macrophages and representative images of immunohistochemistry staining for macrophages (F4/80‐positive cells) in gonadal adipose tissue of HF‐fed WT and TNF ^−/−^ mice. Data were derived from two independent cohorts of mice, where *n* = 5–9 mice per group. Each data point indicates a single mouse. Two‐way ANOVA with Tukey's post test was performed for *A* and *B*, where inset *P* values represent main effects and bars with different letters indicate significant differences. Significance was assessed by Mann‐Whitney test for *C* to *E*. Data in *C* to *E* are shown as bar graphs with mean +/− standard deviation. **P ≤* *0.01, ***P ≤* *0.001.

**Figure 5:**
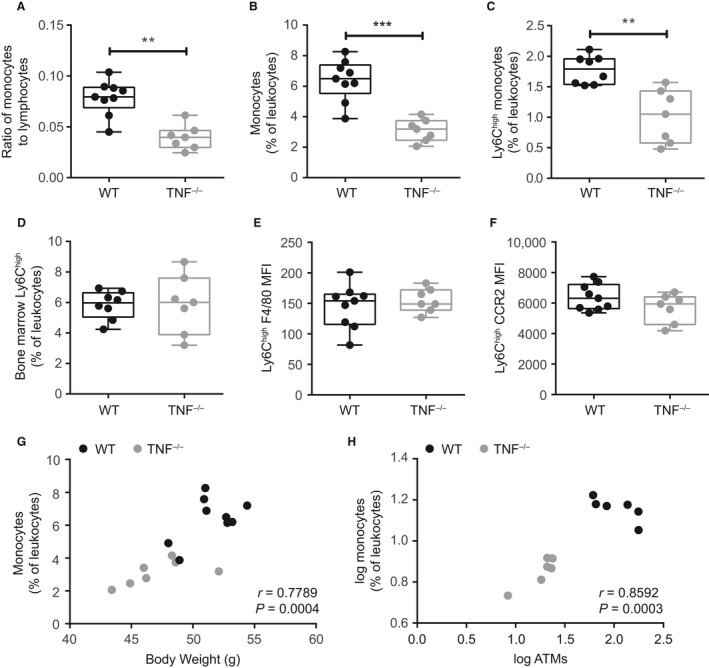
TNF contributes to the egress of monocytes during obesity. Monocyte populations were compared in TNF ^−/−^ and WT male mice allocated to HF diet for 24 weeks. (A) proportion of circulating monocytes to lymphocytes. (B and C) total monocytes and Ly6C^high^ monocytes as a proportion of total leukocytes (CD45^+^ cells). (D) bone marrow Ly6C^high^ monocytes expressed as a proportion of leukocytes. (E and F) circulating Ly6C^high^ surface expression of F4/80 and CCR2. (G) correlation of monocyte prevalence and body weight. (H) correlation of monocyte prevalence and adipose tissue macrophages (ATMs). Data in A to E are representative of two independent cohorts of HF‐fed mice (*n* = 7–9/genotype). Each dot is a mouse. Statistical significance determined by Mann‐Whitney tests. Data in A to E are presented as box and whiskers plots, minimum to maximum, where the center line represents the median. Correlations for G‐H were determined by Pearson’s tests. **P ≤* *0.01, ***P ≤* *0.001. MFI – Geometric Mean Fluorescence Intensity.

We used TNF^−/−^ mice to examine the role of TNF in mediating obesity‐associated changes to inflammatory Ly6C^high^ monocytes. Consistent with previous studies (Uysal et al. 1997; Ventre et al. 1997; Koulmanda et al. 2012), we initially confirmed that TNF^−/−^ mice are partially protected from a HF diet‐induced increase in body mass, as well as insulin resistance assessed by HOMA‐IR (Fig. 4A and B). Lower insulin resistance was due to lower fasting serum insulin rather than changes in blood glucose in HF‐fed TNF^−/−^ mice (Fig. 4C and D). We next showed that HF diet‐fed TNF^−/−^ mice had significantly fewer adipose tissue‐resident macrophages compared to HF diet‐fed WT mice (Fig. 4E). When we examined peripheral monocyte populations, we found there was a twofold reduction in the proportion of circulating monocytes relative to lymphocytes in HF‐fed TNF^−/−^ mice (Fig. 5A). This difference in the ratio of monocytes/lymphocytes in HF‐fed WT mice versus HF‐fed TNF^−/−^ mice is analogous to the ratio of monocytes/lymphocytes in HF‐fed WT mice compared to chow‐fed WT mice (Fig. 1B). Accordingly, total and Ly6C^high^ monocytes were lower in the circulation of HF‐fed TNF^−/−^ mice compared to HF‐fed WT mice (Fig. 5B and C), though Ly6C^high^ monocyte prevalence was similar in HF‐fed WT and TNF^−/−^ mouse bone marrow (Fig. 5D). No differences were observed in circulating Ly6C^high^ monocyte maturity (Fig. 5E) or CCR2 expression between HF‐fed WT and TNF^−/−^ mice (Fig. 5F). These data indicate that TNF contributes to obesity‐associated changes in circulating Ly6C^high^ monocyte prevalence in addition to hyperinsulinemia and macrophage accumulation in metabolic tissues. Indeed, we observed a strong positive correlation in these HF‐fed mice between circulating monocytes and body weight, as well as circulating monocytes and macrophage accumulation in adipose tissue (Fig. 5G and H).

### Ly6C^high^ monocytes correlate with insulin during obesity

**Figure 6:**
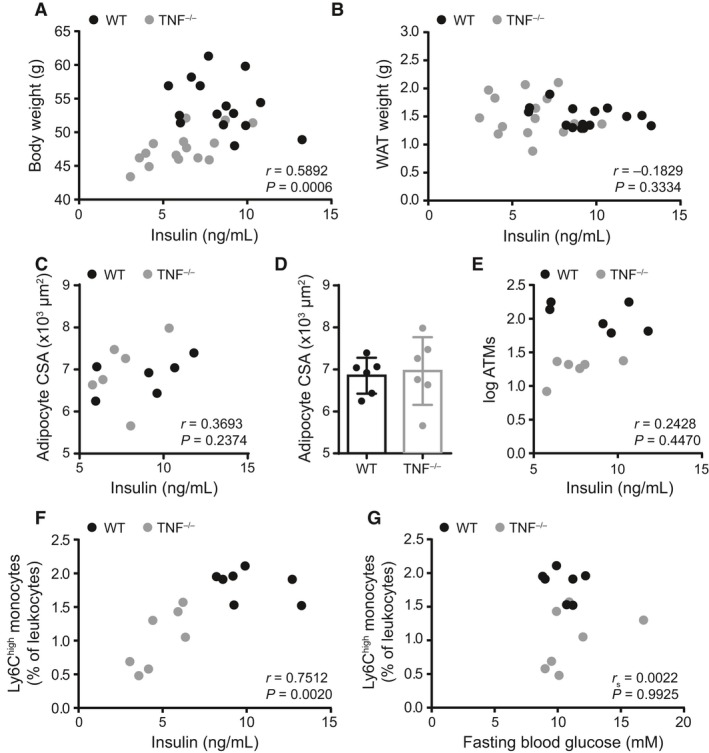
Circulating inflammatory monocytes may predict insulin during obesity. Correlations in HF‐fed TNF^−/−^ and WT mice. (A) correlation of body weight and fasting insulin. (B) correlation of fasting blood insulin and epididymal adipose tissue weight (WAT). (C) correlation of fasting blood insulin and adipocyte cross‐sectional area (CSA). (D) quantification of adipocyte CSA in HF‐fed WT and TNF^−/−^ mice. (E) correlation of fasting blood insulin and adipose tissue macrophages (ATMs). (F) correlation of fasting blood insulin and the prevalence of circulating Ly6C^high^ monocytes in all HF‐fed mice. (G) correlation of fasting blood glucose and the prevalence of circulating Ly6C^high^ monocytes in all HF‐fed mice. Data in A and B are from two independent cohorts of HF‐fed mice (*n* = 7–9/genotype). Data in C–E are from a subset of HF‐fed mice from those cohorts (*n* = 6/genotype). Data in F and G are from one cohort of HF‐fed mice (*n* = 7/genotype). Each dot is a mouse. Data in *D* are shown as a bar graph with mean ± standard deviation. Correlations for A–C and E–G were determined by Spearman or Pearson's tests and Mann‐Whitney U test was used to assess D.

The relationship between adiposity and insulin sensitivity is complicated by many factors, including the lipid storage location (Wagenknecht et al. 2003; Muller et al. 2012). In mice, the mass of specific fat pads and adipocyte cross‐sectional area have previously been shown to mark specific aspects of diet‐induced adiposity (Weisberg et al. 2003; Chusyd et al. 2016). We assessed if body weight, gonadal adipose tissue mass, or adipocyte hypertrophy (i.e., cross‐sectional area of adipocytes), correlated with serum insulin levels in HF‐fed WT and TNF^−/−^ mice (Fig. 6A–C). We combined HF‐fed WT and TNF^−/−^ data and observed that there was a positive correlation between total body weight and insulin. In contrast, neither epididymal adipose tissue mass nor adipocyte cross‐sectional area, as markers of adiposity, correlated with fasting blood insulin. In fact, adipocyte cross‐sectional area was not different in the gonadal fat pads of HF‐fed WT and TNF^−/−^ mice (Fig. 6D). Given the importance of adipose tissue macrophages in driving obesity and metabolic dysfunction (Olefsky and Glass 2010; Osborn and Olefsky 2012), and our observations of the effects of HF diet on the prevalence of circulating monocytes, we next examined whether macrophage and monocyte characteristics related to insulin. The number of adipose tissue macrophages did not correlate with fasting blood insulin (Fig. 6E). However, the prevalence of blood Ly6C^high^ monocytes positively correlated with fasting blood insulin (Fig. 6F and Table 1). Given the direct correlation of insulin and blood Ly6C^high^ monocytes it was logical that we also found an association between Ly6C^high^ monocytes and HOMA‐IR (Table 1). Correlations were also identified between total, Ly6C^−^ and Ly6C^int^ monocytes and fasting blood insulin. We did not observe an association between Ly6C^high^ monocytes and fasting blood glucose (Fig. 6G and Table 1). These data indicate that there is a direct association between serum insulin and the prevalence of circulating immature inflammatory Ly6C^high^ monocytes rather than markers of adiposity such as fat pad mass or adipocyte size in obese male mice.

**Table 1:** Correlations of monocyte populations with body weight and metabolic parameters in obese male mice

	Total monocytes	Ly6C^−^	Ly6C^int^	Ly6C^high^
Body weight	***r*** ** = 0.7790** ***P*** ** = 0.0004**	***r*** ** = 0.6942** ***P*** ** = 0.0029**	***r*** ** = 0.7423** ***P*** ** = 0.0010**	***r*** ** = 0.7526** ***P*** ** = 0.0008**
Fasting blood glucose	*r*_s_ = −0.07517 ***P*** = 0.7729	*r*_s_ = −0.02063 ***P*** = 0.9322	*r*_s_ = −0.2137 *P* = 0.4173	*r*_s_ = 0.0022 *P* = 0.9925
Fasting blood insulin	***r*** ** = 0.6861** ***P*** ** = 0.0067**	***r*** ** = 0.5680** ***P*** ** = 0.0341**	***r*** ** = 0.6418** ***P*** ** = 0.0134**	***r*** ** = 0.7247** ***P*** ** = 0.0034**
HOMA‐IR	***r*** ** = 0.6076** ***P*** ** = 0.0212**	*r* = 0.5070 *P* = 0.0643	*r* = 0.4968 *P* = 0.0707	***r*** ** = 0.7512** ***P*** ** = 0.0020**

Text in bold denotes statistical significance at *P* < 0.05.

## Discussion

In this study we examined the effects of diet‐induced obesity on monocyte prevalence and phenotype. We found that our model of long‐term diet‐induced obesity elevates circulating leukocytes and results in an expansion of peripheral monocytes. We identified that alterations to Ly6C^high^ monocyte phenotype during obesity are TNF‐dependent, begin in the bone marrow, and lead to a higher prevalence of circulating immature and migration‐primed inflammatory Ly6C^high^ monocytes that positively correlate with serum insulin, linking peripheral immune function with endocrine dysregulation.

Monocytosis, an increase in peripheral blood monocytes, has been recognized for many years to coincide with inflammation (Dutta and Nahrendorf 2014), and it has been more recently recognized to contribute to chronic low‐grade inflammation during obesity (Schmidt et al. 1999; Ford 2002; Kullo et al. 2002). We observed a decrease in Ly6C^high^ monocytes in the bone marrow and an increase in this population in circulation in HF diet‐fed obese male mice that are well known to have mild hyperglycemia and hyperinsulinemia. Circulating monocytosis, and a particular elevation of Ly6C^high^ monocytes, has also been reported in Ob/Ob mice (Nagareddy et al. 2014). Ly6C^high^ mouse monocytes are comparable to human classical (CD14^+^CD16^−^) and intermediate (CD14^+^CD16^+^) monocytes (Gordon and Taylor 2005). Classical monocytes both in circulation and adipose tissue increase with obesity (Wouters et al. 2017), and their activation has also been reported in diabetics (Cipolletta et al. 2005). An increase in intermediate monocytes in obese humans has been linked to an increased risk of subclinical atherosclerosis and cardiovascular events (Seidler et al. 2010; Poitou et al. 2011; Rogacev et al. 2012; Ziegler‐Heitbrock 2015), as well as insulin resistance (Krinninger et al. 2014). Expansion of the circulating intermediate monocyte population has also been reported in T1D and T2D patients (Mysliwska et al. 2012; Terasawa et al. 2015; Ren et al. 2017).

In addition to assessing circulating monocyte prevalence, we assessed how obesity affected their progression toward a mature tissue macrophage phenotype by examining their expression of F4/80, a surface marker related to maturity. Our observations of decreased F4/80 expression on the surface of Ly6C^high^ monocytes in bone marrow and blood of HF‐fed mice suggest that these monocytes are less mature. These immature monocytes are known to migrate to tissues in the context of infection via the CCR2/CCL2 chemotactic axis and thus may be primed for migration into metabolic tissues (Weisberg et al. 2006; Tsou et al. 2007; Ito et al. 2008). There were lower Ly6C^high^ monocytes in the bone marrow and higher Ly6C^high^ monocytes in blood, indicating that diet‐induced obesity may alter egress of inflammatory monocytes from the bone marrow into circulation.

We have previously demonstrated a role for TNF in mediating dysfunction of monocyte development and function with aging, as well as increasing susceptibility to infection (Puchta et al. 2016). In addition to their association with increased circulating inflammatory CD14^+^CD16^+^ intermediate monocytes, high levels of circulating TNF are characteristic of obesity as well as chronic inflammatory conditions. Anti‐TNF therapies used to treat rheumatoid arthritis can decrease risk of developing T2D (Klaasen et al. 2011; Solomon et al. 2011; Gremese et al. 2013). We expanded on the pleiotropic role of this pro‐inflammatory cytokine by demonstrating its involvement in inflammatory Ly6C^high^ monocyte egress from the bone marrow into circulation and accumulation of macrophages in metabolic tissues during obesity. TNF may induce NLRP3 inflammasome priming and activation (Alvarez and Munoz‐Fernandez 2013; McGeough et al. 2017). Our data illustrating the importance of TNF in obesity‐associated monocytosis agree with that of Nagareddy and colleagues, who found that obesity‐associated changes in adiposity and monocytosis are dependent on the NLRP3 inflammasome, and in particular NLRP3‐dependent production of IL‐1*β* (Alvarez and Munoz‐Fernandez 2013; Nagareddy et al. 2014; McGeough et al. 2017). The changes we observed to monocyte phenotype in TNF^−/−^ mice were coincident with attenuation of hyperinsulinemia and insulin resistance and a slight reduction in body mass. Lower insulin levels underpinned lower insulin resistance (measured by HOMA‐IR) in the HF‐fed TNF^−/−^ mice. This was expected as TNF is known to promote insulin resistance during obesity (Uysal et al. 1997; Ventre et al. 1997; Hivert et al. 2010; Koulmanda et al. 2012), but this mouse model also provided a unique model to correlate insulin and inflammatory monocytes during diet‐induced obesity. Deletion of TNF reduced accumulation of macrophages in adipose tissue despite similar levels of adipocyte hypertrophy during HFD‐fed feeding. Therefore, our data show that TNF is a key regulator of diet‐induced changes to monocyte/macrophage‐driven inflammation in obesity, and that TNF can contribute to metaflammation in a manner that is independent of adipocyte hypotrophy and regulation of glucose. These data also reinforced the concept that obesity‐induced changes in inflammatory monocytes correlated with insulin rather than markers of adiposity such as adipocyte size.

Our data indicated that tissue‐specific effects of the Ly6C^high^ monocytes and/or metabolic tissue changes predicted the development of some aspects of insulin resistance, which led us to examine the association of monocyte phenotype and insulin levels. Comparisons of blood monocyte characteristics and circulating insulin levels in HF‐fed WT and TNF^−/−^ mice during diet‐induced obesity demonstrated that circulating immature inflammatory Ly6C^high^ monocytes were more strongly associated with serum insulin levels compared to indices of adiposity, total body weight, and other monocyte populations in male mice. Circulating Ly6C^high^ monocytes appear positioned to predict or propagate hyperinsulinemia or insulin resistance during diet‐induced obesity. A recent publication examining the role of CX3CR1 and Gr1^low^ monocytes (equivalent to our Ly6C^−^ monocyte population) in diet‐induced obesity, by using female mice deficient for CX3CR1 in blood or hematopoietic compartments, reported a negative association between Gr1^low^ monocytes and HOMA‐IR (Béliard et al. 2017). Our data in male mice show an association between Ly6C^−^ monocytes and insulin levels rather than HOMA‐IR. Our data were determined through modulation of TNF‐related inflammation in obesity rather than directly modifying monocytes, which may explain this discrepancy. In addition, there are likely sex‐specific differences that alter monocyte characteristics in obesity that warrant further study.

Hyperglycemia and hyperinsulinemia have both previously been shown to alter monocyte epigenetic programming and function in conditions of infection and stress (Xiu et al. 2014), and it has recently been reported that insulin signaling in obesity has a significant role in mediating adaptive T cell inflammatory responses (Tsai et al. 2018). Consequently, the positive correlation that we observed of an increasing prevalence of inflammatory Ly6C^high^ monocytes with increasing insulin resistance in male mice suggested insulin may mediate phenotypic changes to Ly6C^high^ monocytes in obesity. We found that raising insulin was not sufficient to alter circulating Ly6C^high^ monocytes in the absence of obesity. In surprising contrast to a previous study (Nagareddy et al. 2013), we found that hyperglycemic Akita^+/−^ mice did not have changes to circulating Ly6C^high^ monocytes. Our Akita^+/−^ mice were assessed at 8 weeks of age whereas the previous study performed measures on mice at 12‐16 weeks of age (Nagareddy et al. 2013). Longer exposure to elevated peripheral glucose may explain this discrepancy. We did confirm that circulating neutrophils were significantly higher in hyperglycemic Akita^+/−^ mice (Nagareddy et al. 2013), and further showed that ongoing low‐dose insulin exposure (i.e., from a slow‐release implant) can reduce the proportion of circulating neutrophils, likely due to the accompanying reduction in blood glucose. We further demonstrated that reduction of peripheral glucose and insulin via antibiotic treatment in obese wild‐type mice does not alter Ly6C^high^ monocyte populations in circulation. We acknowledge in vivo manipulation of glucose and insulin is interdependent. Nevertheless, our data suggest that elevated blood glucose or insulin in the absence of cellular mediators, hormones, hyperlipidemia, and other factors that accompany obesity, are insufficient to alter monocyte prevalence or measurements of maturity (F4/80) or chemotactic potential (CCR2). We cannot exclude the possibility that dyslipidemia or hyperleptinemia may also contribute to the changes in monocyte maturity and phenotype that we observed with HF diet (Desai et al. 2017; Rahman et al. 2017; Short et al. 2017). A significant limitation of our data is that our WT and TNF^−/−^ experiments were not conducted in littermate mice. Although metabolic data were consistent with previous data, our study design cannot rule out the possibility that differences in genetic background, microbiota composition, or husbandry may have influenced the results (Hotamisligil et al. 1995; Uysal et al. 1997; Ventre et al. 1997). The use of non‐littermate mice is a weakness of this study to provide a direct role of TNF, but the association of inflammatory monocytes with insulin may span a diverse genetic background and should be further investigated in humans. Our current data support a model where obesity‐related cellular mediators alter monocyte characteristics, which contribute to cellular inflammation and hormone regulation within metabolic tissues. Our data suggest directionality in the relationship between insulin and monocyte changes during obesity, where diet‐induced changes in Ly6C^high^ monocytes predict insulin and insulin resistance, but neither glucose, insulin, nor insulin resistance appears to alter these inflammatory monocytes during obesity.

Monocyte prevalence and phenotype have been proposed as biomarkers in cardiovascular disease and chronic inflammatory disorders (Yang et al. 2014; Chara et al. 2015; Meeuwsen et al. 2017; Loukov et al. 2018). Our data links monocyte/macrophage phenotype to changes in whole body insulin regulation due to diet‐induced obesity and suggests that monocyte characteristics in obese individuals could serve as predictive biomarkers of diabetes risk and may represent a mechanism linking inflammation and insulin regulation. Modulation of low‐grade inflammation through selectively targeting monocyte populations in obese individuals may therefore improve insulin sensitivity. Examining the role of all monocyte subsets in driving peripheral and tissue‐specific metainflammation will improve our understanding of the development of hyperinsulinemia, insulin resistance, and type 2 diabetes in obese individuals.

## Conflict of Interest

The authors have no conflicts of interest, financial, or otherwise, to be declared.
